# Sleep of Animals and Man

**Published:** 1843-03

**Authors:** 


*Sleep of Animals and Man.*—Most animals sleep more than man; some indeed for months—as the hibernating tribes of bats, dormice, marmots and bears. Cats and dogs would seem to have the faculty at will, as have some idiots and persons of a low order of intellect. The ideas or impressions upon their minds are so feeble or so few, or are made at such long intervals that succession is lost for want of continuity; hence the organ retains imperfectly, and but for an instant, the image which the external senses have presented to it; weariness supervenes, unconsciousness follows, and lastly, sleep, as a necessary consequence of inanition, is induced. It is observed, however, that monkeys do not sleep as much as other animals. Whence is this apparent deviation from the ordinary law of nature affecting animals? Is a monkey a reasoning animal? Observe a dog chained: he twists his chain, shortens it, and cuts himself off from his platter. Does he seek to untwist it, to restore the links to their wonted extension? No; he continues tugging and howling till some friendly hand frees him from his toils, and restores him to his former range. But how is it with the monkey under similar difficulties? Why, he deliberately untwists the chain which he cannot sunder, and hence evinces something like reason. Is the sleeplessness of monkeys then a proof of reason? We think so. But infants are frequently sleepless? Yes; but never in a state of health. Restlessness in them is always an indication of hunger, or a symptom of disease. The absence of sleep cannot be long sustained. Damiens slept on the rack; Luke in his iron crown; and a battalion of infantry have been known to slumber during a march! Muleteers frequently sleep on their mules, post-boys on their horses, and seamen "on the high and giddy mast." "Massa call you," said a negro to his comrade who had fallen asleep near him; "Sleep has no massa," replied the wearied boy; and he was right. We may bear the privation of fire, food, and even drink, longer than we can the want of sleep.—*Dr. Binn's Anatomy of Sleep.*

